# Drug-target binding affinity prediction based on power graph and word2vec

**DOI:** 10.1186/s12920-024-02073-5

**Published:** 2025-01-13

**Authors:** Jing Hu, Shuo Hu, Minghao Xia, Kangxing Zheng, Xiaolong Zhang

**Affiliations:** 1 School of Computer Science and Technology, Wuhan University of Science and Technology, Wuhan, 430065 Hubei China; 2 Hubei Province Key Laboratory of Intelligent Information Processing and Real-Time Industrial System, Wuhan, China; 3 Institute of Big Data Science and Engineering, Wuhan University of Science and Technology, Wuhan, Hubei China

**Keywords:** Drug-target affinity, Power graph, Word2vec, Graph neural network, Drug retargeting

## Abstract

**Background:**

Drug and protein targets affect the physiological functions and metabolic effects of the body through bonding reactions, and accurate prediction of drug-protein target interactions is crucial for drug development. In order to shorten the drug development cycle and reduce costs, machine learning methods are gradually playing an important role in the field of drug-target interactions.

**Results:**

Compared with other methods, regression-based drug target affinity is more representative of the binding ability. Accurate prediction of drug target affinity can effectively reduce the time and cost of drug retargeting and new drug development. In this paper, a drug target affinity prediction model (WPGraphDTA) based on power graph and word2vec is proposed.

**Conclusions:**

In this model, the drug molecular features in the power graph module are extracted by a graph neural network, and then the protein features are obtained by the Word2vec method. After feature fusion, they are input into the three full connection layers to obtain the drug target affinity prediction value. We conducted experiments on the Davis and Kiba datasets, and the experimental results showed that WPGraphDTA exhibited good prediction performance.

## Background

In the field of drug-target interactions, the traditional way of research is wet experiments. However, traditional wet experiments are inefficient, expensive and time-consuming [1, 2]. According to statistics, it takes an average of 10 to 15 years to develop each new drug. At the same time, drug-related regulations are improving, it is becoming increasingly difficult to obtain approval for drugs, and the time and cost of new drug development are rising. In addition, traditional methods need to be coupled with high-throughput screening assays to detect biological activity between drugs and proteins, making drug development more expensive and time-consuming [3, 4]. Predicting drug-target interactions (DTIs) by finding new uses for already approved drugs [5] can reduce development costs and shorten drug development cycles to some extent [6].

Drug-target interaction refers to the action of a drug molecule on a target protein and the bonding reaction with the target protein [7], thus affecting the pharmacological action of the protein to achieve phenotypic effects, which is a prerequisite for the drug to have an effect. Predicting drug-target interactions allows researchers to discover new drug targets in the most efficient way, thus saving time and money while reducing the potential for future adverse reactions and side effects. Predicting drug-target interactions by computer allows rapid prediction of the likely effects of new drugs and helps to screen promising compounds more efficiently.

Methods for predicting drug-target relationships can usually be divided into two categories. One is to predict drug-target interactions based on binary classification, and the other is to analyze drug-target affinity (DTA) by regression methods. In binary classification-based DTI prediction studies, researchers initially used machine learning methods, but have now begun to use a wide range of deep learning techniques, including restricted Boltzmann machines [8], deep neural networks [9, 10] (DNNs), stacked autoencoders [11, 12], and deep belief networks [13] (DBNs). However, binary classification ignores an important piece of information about drug-target interactions, i.e., binding affinity. Affinity is a characteristic function of the relative states between the candidate drug molecule, the target molecule epitope and the candidate drug molecule-target molecule binding during the reversible reaction. Drug-target binding affinity reflects information on the strength of the interaction between drug-target pairs. For drug development, drug-target affinity is one of the key indicators for determining drug efficacy, and accurate prediction of the affinity between drug candidate molecules and targets is a critical step in understanding the principle of action of drug candidates.

The least squares (KronRLS) method based on Kronecker regularization has shown impressive results among the early machine learning algorithms to estimate the binding affinity of drug targets [14, 15]. This method calculates the similarity scores between drug and protein targets and represents them as a similarity score matrix using the Smith-Waterman (S-W) algorithm [16] and PubChem structural clustering tool. In addition, there is a well-known machine learning method, the gradient boosting-based method SimBoost [17]. This method uses feature engineering of compounds and proteins to represent DTI, using similarity-based information and network-based features to predict drug target binding affinity.

A popular approach for predicting drug-target binding affinity is to feed the sequence of the target protein and drug (1D representation) into the deep learning model after it has undergone continual improvement. For example, DeepDTA [18] uses two convolutional neural network (CNN) blocks to learn drug and protein representations separately, and then connects these learned representations and inputs them into a fully connected layer to predict drug-target binding affinity scores. The WideDTA [19] model is based on an extension of the DeepDTA model, WideDTA uses four text-based information sources, i.e., drug SMILES (Simplified Molecular Input Line Entry System) molecules, protein sequences, protein structural domains and motifs(PDM), and ligand maximum common substructures(LMCS) [20], and this model combines and filters these four types of information into two CNN blocks to predict binding affinity. GraphDTA [21] used graph neural networks to extract drug features and combined them with protein features extracted by CNN to achieve good results in predicting drug-target affinity. Li [22] et al. proposed a co-regularized variational autoencoder (Co-VAE) capable of predicting drug and target affinities based on drug structure and target protein sequences. The model uses two variational autoencoder (VAEs) to generate drug strings and target sequences, respectively, and uses the co-regularized part to generate binding affinities. It was theoretically demonstrated that the Co-VAE model is a lower bound for maximizing the joint likelihood of drugs, proteins and their affinities.

The information of physicochemical properties and molecular structures of drugs and proteins have been used in different models. Meanwhile, some algorithms based on machine learning and deep learning [23] have been developed to gain insight into the strength of drug-target interactions. Currently, graph neural networks have shown good performance in drug-target affinity prediction, which also provides some inspiration for the exploration in this paper.

In this paper, we propose a drug target affinity prediction model based on power graphs and Word2vec. It is based on the one-dimensional amino acid sequence of the target protein and the one-dimensional SMILES sequence of the drug molecule, and uses RDKit [24] to encode the drug SMILES into a two-dimensional molecular graph, then uses the power graph representation to obtain the topological information of the graph, and divides the amino acid sequence into sentences consisting of biological words containing contextual information, and converts the sentences by a pre-trained Word2vec dictionary converted into an embedding matrix [25]. The drug-target features are then extracted using a deep neural network and connected followed by a fully connected layer to predict the binding affinity. In this paper, experiments were conducted on two benchmark datasets, Davis and Kiba, and the results show that the present model exhibits better prediction accuracy compared to other advanced interaction prediction methods for the same dataset.

## Methods

### Model overview

We propose a power graph and word2vec based model WPGraphDTA for predicting drug target binding affinity. The model has two functional modules, the first module encodes drug SMILES as a 2D (two-dimensional) molecular graph, uses power graph representation to obtain topological information of the graph, and inputs it into a graph convolutional neural network to extract drug features. The second module encodes amino acid sequences into an embedding matrix using word2vec, which is input into a CNN block to obtain local chemical information of the target/protein.

### Drug representation

Drugs are usually small molecular compounds compared to the large and complex structures of proteins. The simplified molecular input line entry specification (SMILES) [26] is used to represent drug molecules. SMILES is able to obtain structural information of compounds, including chirality, molecular rings, chemical bonds, carbon chain branches and constituent elements. The strength of SMILES being used to represent drug molecules lies in its uniqueness, which ensures that each drug molecule has a unique structural sequence representation and that each sequence has a unique drug molecule corresponding to it, which facilitates the extraction of valid drug molecule composition and structural features from one-dimensional sequence data.

Smiles can be converted into a two-dimensional structure diagram g = (v, e). Among them, V represents the atoms of the drug molecule, which is the node in the two-dimensional graph. E means the chemical bond of the drug molecule, i.e., the edge in the two-dimensional structure graph, and it usually appears in the form of an adjacent matrix. In the process, two tools were mainly used, namely RDKit and DeepChem [27]. Each atom in the molecular graph is expressed as the corresponding feature vector. The information contained in this vector includes atomicity, total hydrogen, atomic symbols, hidden values of atoms and whether they exist. DeepChem can obtain atomic symbols, and RDKit can obtain the remaining four characteristics. Then convert the drug SMILES molecule into an adjacent matrix A, and combine A, A^2^, A^3^ and the neural network to obtain the characteristics of the drug.

### Protein representation

Typically, amino acid sequences are used to express protein targets (for example, MKKHHDSRREQ…). This model uses word2vec to encode the amino acid sequence into an embedded matrix and pass it as input to the CNN block to obtain the local chemical information of the target/protein. In the biological environment, a single amino acid is usually meaningless. This model uses a fixed-length N-gram split method to divide the protein sequence into meaningful “biological words”. The sequence here refers to the input protein sequence (instead of complete sequences) with a fixed length after pre-processing (long interception, Short with 0 filling). The protein sequence is split up into N-Gram sequences using the fixed-length N-Gram approach, and each N-Gram is regarded as a “biological word”. Compared with natural encoding, it can reflect the context information of the amino acid sequence, so it can obtain more comprehensive information of the target protein.

There are usually 20 amino acids that work in the human body, so the maximum number of N-gram is 20^N^. After weighing the feasibility and vocabulary of the model training [28], we define *N* = 3. Specifically, a protein sequence is given L = {"MKKFD"}, and the sequence is divided using the fixed 3-g division method. A biological word made up of 3 amino acids is called a 3-g. The result of the segmentation is L = {"MKK", "KKF", "KFD"}. For each biological word, it was mapped to an embedding vector by finding a pre-trained embedding dictionary [29] containing 9048 words obtained from Swiss-Prot with 560118 manually annotated sequences. Through 3-g, each protein sequence is converted into a matrix, each of which contains an embedded representation of a biological word. Then input this matrix into CNN to extract the context information of the target protein.

### WPGraphDTA model structure

In most of the previous drug target affinity studies, the one-hot approach was mostly used to encode drugs and proteins, but later it was found that the performance of the model was significantly improved after representing drug molecules as a graph. We need to find a method that can accurately extract drug features based on graph information, and graph convolutional neural networks have shown good performance in this regard and are therefore applied in this model. Another group of methods utilizing information networks can also improve the accuracy of predictions [30–32]. We also introduced the idea of power graph used to obtain the topological information of the graph.

**Fig. 1:**
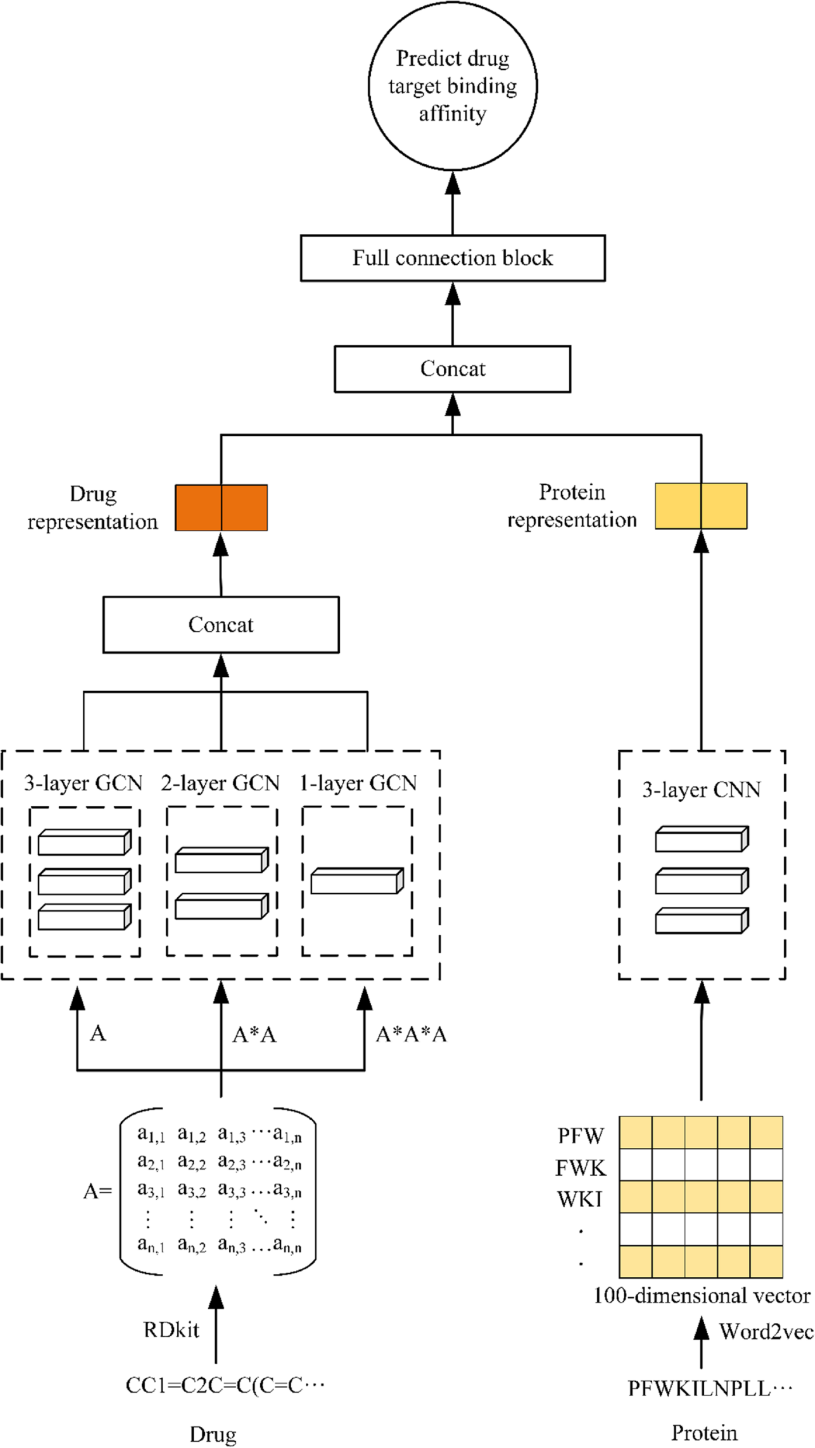
Model architecture of WPGraphDTA

In this paper, a drug-target affinity prediction model based on power graph and word2vec is proposed. Figure 1 shows the architecture of the model. The model inputs were the amino acid sequences of proteins and the SMILES sequences of drug molecules. The model is divided into three modules, which are the protein feature extraction module, the drug molecule feature extraction module, and the affinity prediction module for drug-target interactions.

For proteins, the protein sequence is a string of ASCII (American Standard Code for Information Interchange) characters that represent amino acids. This model uses word2vec to encode amino acid sequences into an embedding matrix, which is used to input CNN blocks to obtain local chemical information of the target/protein. Specifically, we first apply a fixed-length N-gram splitting method to segment protein sequences into meaningful “bio-words”, map them to an embedding vector by finding a pre-trained embedding dictionary, and then convert each protein sequence into a matrix in which each row is embedded with a bio-word via a 3-g. The matrix is fed into a 3-layer CNN to learn the input features, in general, the more convolutional layers the better the feature extraction ability, but the more convolutional layers are not better, because as the number of convolutional layers increases it will lead to the occurrence of problems such as overfitting, so this model finally chooses to use a 3-layer CNN to learn the input features, and finally after the maximum pooling layer to get the feature representation of the input proteins.

For drugs, the input is the SMILES sequence of the drug molecule, and we use the RDkit tool to convert the SMILES sequence of the drug molecule into the corresponding molecular graph representation, and we use five atomic features adapted from DeepChem to characterize the nodes in the graph, where each node is represented as a multi-dimensional binary feature vector. In order to learn information about drug molecules at a deep level, we introduced the idea of power graph in the drug feature extraction stage. The features of the power graph are extracted by the GCN (Graph Convolutional Network) module, and finally the obtained features are concatenated to obtain the final drug feature representation after the maximum pooling layer.

To predict the drug-target affinity score, it is important to understand the interaction of each node with its neighboring nodes [33]. When representing complex graph data, considering only connectivity relationships between directly adjacent nodes may not fully capture the overall features of the graph. By adding multi-hop connectivity relationships, more distant correlations between nodes can be considered, which in turn provides a more comprehensive representation of graph-level features. This is particularly useful for graph representations of drugs because not only direct interactions between compounds are considered, but also indirect correlations between them, such as common functional and metabolic pathways, can be taken into account. Therefore, considering multi-hop connectivity relationships between nodes when constructing graph-level feature representations can provide richer information to better characterize the overall features and interactions of graph data. This helps to understand the relationships between complex graph data, networks, and drug compounds.

**Fig. 2:**
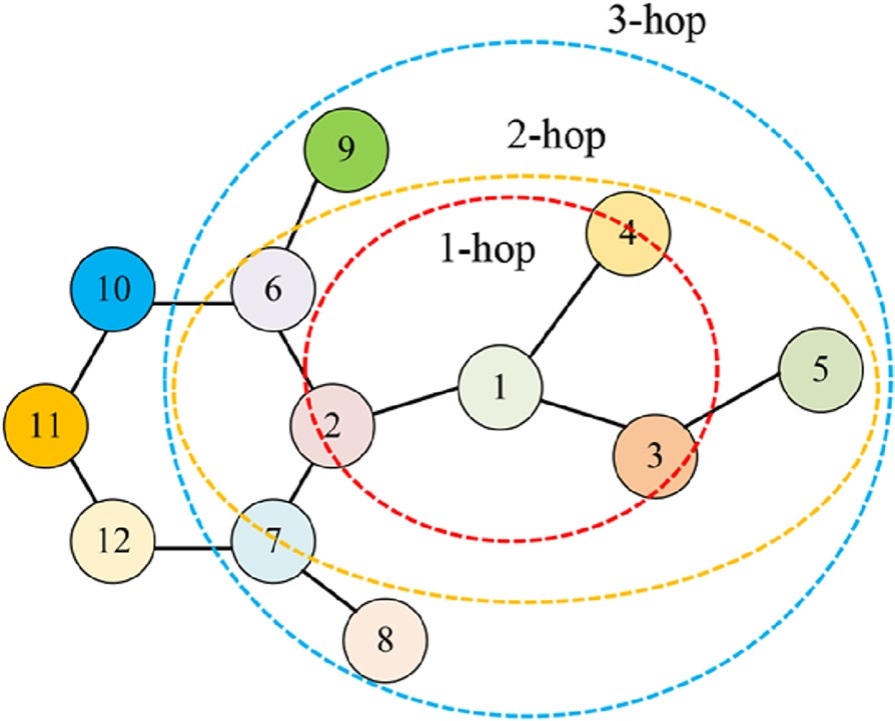
Molecular graph representation of 1-hop, 2-hop, and 3-hop Power Graphs

In order to learn information about drug molecules in a deeper level, this paper attempts a power graph-based drug feature extraction strategy. In the molecular graph, each node v is connected to each node u in its neighborhood R(v) by edges. also, for each node w in R(u), if w is not in R(v), then the shortest path distance between w and v is 2, i.e., they are two hops away from each other. If v is connected to all these nodes, i.e., to nodes that are two hops away from it, then this graph can be called a power of 2 graph, which is usually denoted by A^2^. Similarly, by increasing the value of the exponent, the number and range of connections can be increased, allowing node v to establish direct or indirect connections with more nodes, enhancing the local accessibility of node v. It is important to note that increasing the index value of the power graph leads to an increase in the size and complexity of the graph. In general, the shortest path when describing the structure of a drug molecule graph is usually no more than 3 hops for the consideration of computational efficiency and practicality. Therefore, increasing the exponent of the power diagram to more than 3 has a very limited improvement on the model performance, but it will consume great computational resources, after weighing, we set the maximum exponent of the power diagram as 3. The molecular graph representation of 1-hop, 2-hop and 3-hop power graphs with node 1 as the center node is shown in Fig. 2.

This model captures the connectivity relationship between graph nodes mainly through three GCN blocks. In general, more GCN layers are not better, and the representation vectors may converge when there are too many layers, and problems such as gradient disappearance, oversmoothing, and overfitting may occur, making it difficult to continue the relevant learning tasks. As shown in Fig. 1, in the first block, three GCN layers are stacked, which are mainly responsible for extracting the features of the power-of-one graph. In the second block, two GCN layers are stacked, which are responsible for extracting the features of the square power graphs. And in the last block, only one GCN is used to extract the features of the cubic power graphs. Finally, the final drug molecule features were obtained by combining the features extracted from the three GCN blocks.

In this paper, we present the specific propagation rules as an example for the first block of GCNs. The adjacency representation (A ∈ R^N*N^) and the node feature matrix (X ∈ R^N*C^, where C is the number of features for each node) produced by the RDKit tool are used as inputs in the first block to calculate the basic propagation rules for each drug compound. To overcome the degree normalization problem of the adjacency representation, the model uses the following method to compute the normalized adjacency representation (A_norm_), as shown in Eq. (1).

1 $$A_{norm} = D^{{ - \frac{1}{2}}} AD^{{ - \frac{1}{2}}}$$

where D ∈ R^N×N^ is the degree matrix representation of A. $D^{{ - \frac{1}{2}}}$ represents the inverse square root of the degree matrix.

The normalized adjacency matrix A_norm_ can be used as a weight matrix in the propagation rules in the GCN model to capture the strength of connections and relationships between nodes. In this way, the propagation rules can use the adjacency matrix and the node feature matrix for information propagation and feature updating.

To make the first block in the GCN workable, the global representation of A generated at layer i of the GCN module (H ∈ R^N*M^) is computed by (2).

2 $$H_{{\mathrm{e}}}^{i} = \sigma \left( {A_{norm} H_{{\mathrm{e}}}^{{\left( {i - 1} \right)}} W^{{\left( {i - 1} \right)}} } \right)$$

where W is the trainable weight, H0 e is the layer i output representation, and σ is the nonlinear activation function.

Similarly, we can obtain the global representations of A2 norm, A3 norm, and the three GCN blocks. Finally, we join the output representations of the three blocks to obtain the final representation of each drug compound.

## Results

### Dataset

We evaluated our model on two publicly available benchmark datasets, Davis and Kiba [34, 35]. These two datasets have been widely used in various previous DTA predictions.

The Davis dataset, which was collected by Davis et al. in 2011, contains 30,056 interactions of 68 drugs and 442 proteins, and its binding affinity is obtained by measuring the Kd values of 68 drugs and 442 proteins. To address the problem of large disparity in the distribution of affinity values, in 2017, He et al. logarithmically transformed the affinity values of Davis into a logarithmic space with a base of 10, and used a new metric pK_d_ to measure the affinity, which was calculated as shown in Eq. (3). The logarithmically transformed Davis dataset affinity values were concentrated between 5 and 11.

3 $${\mathrm{p}}K_{d} = - \log_{10} \left( {\frac{{K_{d} }}{{1{\mathrm{e9}}^{{}} }}} \right)$$

The Kiba dataset contains binding affinities for 229 proteins and 2111 drugs, and the Kiba dataset is derived from the Kiba method, which combines the biological activities of kinase inhibitors from different sources. To reconcile the consistency between the different information, the KIBA dataset introduces the KIBA score, which integrates statistical information on Kd, Ki (Inhibition Constant) and IC50 (Half-Maximal Inhibitory Concentration) into a single bioactivity score for drug-target interactions. The original Kiba dataset contained 467 targets and 52,498 drugs. He et al. screened the Kiba dataset in 2017 to retain only drugs and targets with interaction numbers more than 10, and thus the benchmark dataset, Kiba, which we now widely use, was obtained, which contains 229 proteins and 2111 drugs, with affinity values ranging from 0.0 to 17.2. Table 1 shows relevant information about the two benchmark datasets.

**Table 1:** Basic information of the benchmark dataset

Dataset	Proteins	Compounds	Interactions
Davis	442	68	30056
Kiba	229	2111	118254

### Evaluation metrics

To assess the effectiveness of the model, two evaluation metrics widely used in regression problems were set in this experiment, namely the mean square error (MSE) and the consistency index (CI) [36]. The consistency index aims to measure the difference between the predicted binding affinity values of two random drug-target pairs and the true values, a larger CI indicates a better prediction by the model and it is calculated as shown in Eq. (4):

4 $$CI = \frac{1}{Z}\sum\limits_{{y_{i}> y_{j} }} {h(p_{{\mathrm{i}}} - p_{j} )}$$

where p_i_ is the predicted value of the larger affinity y_i_, p_j_ is the predicted value of the smaller affinity y_j_, h(x) is the step function [37], and Z is a normalization constant that maps values to the interval [0,1]. In general, h(x) is defined as shown in Eq. (5):

5 $${\mathrm{h}}(x) = \left\{ {\begin{array}{*{20}c} {1,x> 0} \\ {0.5,x = 0} \\ {0,x < 0} \\ \end{array} } \right.$$

Mean square error (MSE) is a measure that reflects the degree of difference between the predicted value and the true value, and the smaller the MSE is, the closer the predicted value is to the true value, which means that the model is more effective, and it is usually calculated using Eq. (6):

6 $$MSE = \frac{1}{n}\sum\limits_{i = 1}^{n} {(P_{i} - } Y_{i} )^{2}$$

where P_i_ and Y_i_ are the predicted and true values of the affinity for the i-th drug target pair and n is the overall number of samples. In general, a larger CI and a smaller MSE demonstrate better model performance.

### Parameter setting

To determine the hyperparameters, the model uses a five-fold cross-validation, where the dataset is disrupted and randomly divided into five equal parts, and one part is selected as the validation set and the remaining four parts are the training set. The model is trained on the four training sets and then validated on the validation set, repeated five times, with each one as the validation set and the remaining four as the training set, to record the average results and evaluate the model performance. Finally, the model trained with the data from the five folds is tested on the benchmark dataset and the final model performance evaluation is obtained. The final hyperparameter settings chosen for our model are shown in Table 2.

**Table 2:** Our model hyperparameter settings

Parameter	Setting
Epoch	500
Protein length	1000
CNN layers	3
Number of power graph blocks	(A, A^2^, A^3^)
Dropout	0.2
Learning rate	0.0005
Batch size	512

### Experimental results

In this paper, we propose a power graph and word2vec based model to predict drug target binding affinity, and to validate the performance of this model, experiments are conducted on Davis dataset and Kiba dataset in this section. We compare our results with KronRLS, SimBoost, DeepDTA, WideDTA and GraphDTA. The experimental results are evaluated using mean square error (MSE) and consistency index (CI), with lower MSE values indicating that the predicted values of the model are closer to the true values, while higher CI values are more consistent with the actual values.

Table 3 compares the performance of the WPGraphDTA model with other benchmark models on the Davis dataset, and it can be seen that our model achieves optimal results in both evaluation metrics, MSE and CI. The MSE of the model in this paper is 0.226, which is 40.3%, 19.8%, 13.4%, 13.7% and 1.3% lower than the MSE of the baseline models KronRLS, SimBoost, DeepDTA, WideDTA and GraphDTA, respectively, and the CI values are improved by 2.8%, 2.6%, 1.9%, 1.0%, and 0.2%, respectively. As can be seen, our model showed good predictive performance on the Davis dataset. Table 4 compares the performance of the WPGraphDTA model with other baseline models on the Kiba dataset, and it can be seen that our model shows good performance on the Kiba dataset and shows the best results on both MSE and CI compared to all other baseline models.

**Table 3:** Performance comparison with other models on Davis dataset

Model	Protein rep	Compound rep	MSE	CI
KronRLS	Smith-Waterman	Pubchem-Sim	0.379	0.871
SimBoost	Smith-Waterman	Pubchem-Sim	0.282	0.872
DeepDTA	1D	1D	0.261	0.878
WideDTA	1D + PDM	1D + LMCS	0.262	0.886
GraphDTA	1D	GIN	0.229	0.893
**WPGraphDTA**	Word2vec	(A, A^2^, A^3^)	**0.226**	**0.895**

**Table 4:** Performance comparison with other models on the Kiba dataset

Model	Protein rep	Compound rep	MSE	CI
KronRLS	Smith-Waterman	Pubchem-Sim	0.411	0.782
SimBoost	Smith-Waterman	Pubchem-Sim	0.222	0.836
DeepDTA	1D	1D	0.194	0.863
WideDTA	1D + PDM	1D + LMCS	0.179	0.875
GraphDTA	1D	GIN	0.139	0.891
**WPGraphDTA**	Word2vec	(A, A^2^, A^3^)	**0.134**	**0.898**

Tables 3 and 4 show that our model shows the best results on both benchmark datasets compared with other benchmark models based on machine learning and deep learning. In particular, we have improved performance on both compared with GraphDTA, which is based on graph neural networks. In GraphDTA, the model uses a GCN block to extract drug feature information and a 1D CNN to extract protein sequence information. In our model, we use a power graph approach to obtain more drug feature information in order to obtain more topological information and use word embedding to encode amino acid sequences into an embedding matrix, so we obtain better performance than GraphDTA.

**Fig. 3:**
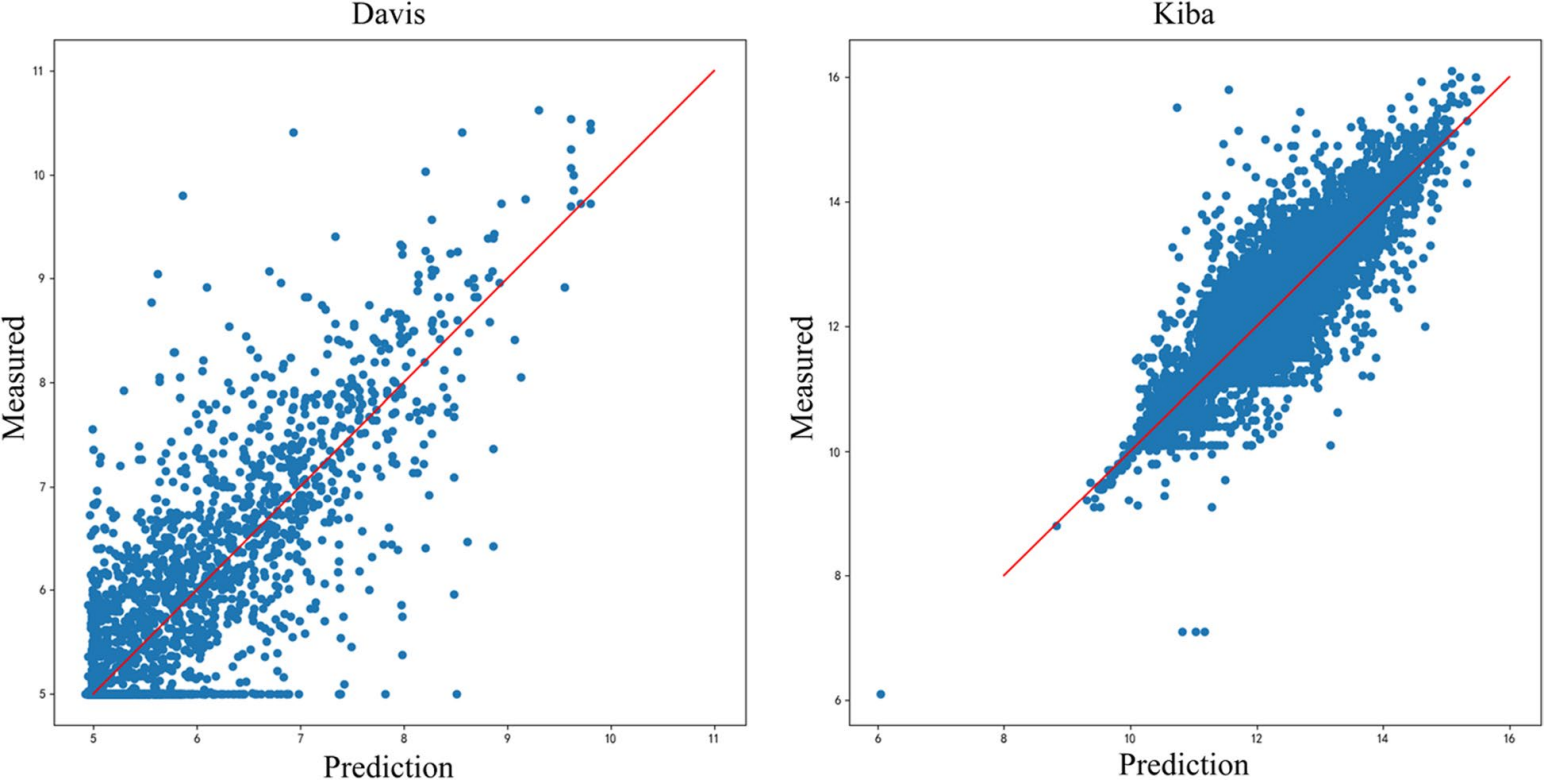
Scatter plot of drug target affinity prediction results of WPGraphDTA on Davis and Kiba datasets

Figure 3 shows the scatter plot of the prediction results of this model on two benchmark datasets, where p is the predicted value and m is the actual value. The closer the predicted and actual values are, the better the model works, i.e., the sample points should fall near the straight line (p = m). From the scatter plot of the prediction results, we can see that the sample points are distributed around the straight line (p = m), which also indicates the good prediction performance of our model.

## Discussion

### Ablation study

To verify the contribution of each functional module in the model to the performance, we conducted ablation experiments on the Kiba dataset for the word2vec and power graph modules, respectively. We replaced word2vec with the traditional one-hot encoding and observed the experimental results as a way to verify the effectiveness of word2vec on the model. Then, we decomposed the power graph block and did separate comparison experiments to observe the contribution of the power graphs to the model.

For the word2vec ablation experiments, this paper chooses to replace the word2vec with protein one-hot encoding, and other factors are controlled unchanged. For the power graph block, three experiments are performed in this section, which are to extract drug molecular map features using only primary, quadratic and cubic power graphs and keeping the protein sequences processed with word2vec unchanged. The KIBA dataset was selected for this experiment and tested for 500 epochs, and the experimental results are shown in Table 5.

**Table 5:** Results of ablation experiments on the Kiba dataset

Model	Protein encoding	Compound rep	MSE	CI
Remove word2vec	One-hot	(A, A^2^, A^3^)	0.216	0.867
Remove (A, A^2^)	Word2vec	A^3^	0.143	0.886
Remove (A, A^3^)	Word2vec	A^2^	0.145	0.889
Remove (A^2^, A^3^)	Word2vec	A	0.137	0.895
**WPGraphDTA**	Word2vec	(A, A^2^, A^3^)	**0.134**	**0.898**

As can be seen from Table 5, the performance of MSE and CI decreased by 38% and 3.6%, respectively, after we replaced the word2vec of the model with One-hot encoding, which shows that the word2vec module has a greater impact on the performance of this model. The theoretical conjecture of this experiment is that using word2vec can extract the contextual information of amino acid sequences, while in One-hot encoding, each amino acid is independent and the contextual information of amino acid sequences cannot be extracted using CNN, and the experimental results also show that the word2vec-based model performs better.

For the power graph module, we compared the WPGraphDTA model using only power graph A, squared power graph A^2^, cubic power graph A^3^, and a combination of (A, A^2^, A^3^). The experimental results show that the power graph module alone is much less effective than the WPGraphDTA model combining (A, A^2^, A^3^), while the inclusion of (A^2^, A^3^) contributes significantly to the lower MSE obtained by the model. The results of the ablation experiments show that both the word2vec module and the power graph module contribute significantly to the performance improvement of the model in this paper.

## Conclusion

In this paper, we propose a power graph and word2vec based drug target affinity prediction method, which transforms drug SMILES sequences into drug molecule graphs and then extracts drug molecule graph features from the power graph module using GCN. For proteins, our model uses a word2vec approach to split protein sequences into meaningful “biological words”, which are then encoded into an embedding matrix and fed into a two-dimensional convolutional neural network for training. We conducted experiments on two benchmark datasets, Davis and Kiba, and the experimental results showed that our model obtained the best results on two evaluation metrics, MSE and CI, compared with other benchmark models, indicating that our model can effectively improve DTA prediction and has good prediction performance.

## Data Availability

The datasets used and analyzed during the current study are available from the corresponding author on reasonable request.

## Abbreviations

DTIs: Drug-target interactions
DTA: Drug-Target Affinity
DNNs: Deep neural networks
DBNs: Deep belief networks
S-W: Smith-Waterman
CNN: Convolutional Neural Network
SMILES: Simplified Molecular Input Line Entry System
Co-VAE: Co-regularized variational autoencoder
VAE: Variational autoencoder
2D: Two-dimensional
ASCII: American Standard Code for Information Interchange
Kd: Dissociation Constant
Ki: Inhibition Constant
IC50: Half-Maximal Inhibitory Concentration
MSE: Mean square error
CI: Consistency index
PDM: Protein structural domains and motifs
LMCS: Ligand Maximum Common Substructures
GIN: Graph isomorphism network
